# Decarbonising the automotive sector: a primary raw material perspective on targets and timescales

**DOI:** 10.1007/s13563-022-00334-2

**Published:** 2022-08-18

**Authors:** Evi Petavratzi, Gus Gunn

**Affiliations:** British Geological Survey, Nicker Hill, Keyworth, Nottingham, NG12 5GG UK

**Keywords:** Battery raw materials, Automotive supply chain, Mining timescale, Supply risk

## Abstract

Decarbonisation of the automotive sector will require increased amounts of raw materials such as lithium, cobalt, nickel and rare earth elements. Consequently, it is crucial to assess whether supply will be able to meet forecast demand within the required timescale. The automotive sector relies on complex global supply chains comprising four tiers. We have developed an integrated timeline from tier 4 (supply of raw materials) through to tier 1, the production of electric vehicles (EVs). Numerous factors, mainly economic, political, social and environmental, influence the duration of tier 4 leading to considerable variation between projects. However, our analysis demonstrates that it commonly takes more than 30 years from initial exploration to EV production. Tier 4, which is often neglected by the automotive industry, may account for 20 years of that period. This suggests that raw material supply is unlikely to match the projected demand from electrification of the automotive sector up to 2030. Reducing the duration of tier 4 will be difficult, although governments and industry can mitigate supply risks in various ways. These include multi-disciplinary international research across the supply chain and the transformation of research findings into policy and best practice. Supply chain convergence, with businesses across the supply chain working to develop long-term plans for secure and sustainable supply, will also be beneficial. In addition, global stakeholders should work together to resolve ESG challenges to supply. All these measures depend on the availability of researchers and industry personnel with appropriate skills and knowledge.

## Introduction

Economic growth, combined with a burgeoning world population, has contributed to significant climate change on a global scale. It has now become urgent for economies to decarbonise in order to limit global warming to 1.5 °C (UNEP 2019). Decarbonisation of transport can provide the biggest reduction in carbon dioxide (CO_2_) emissions, in the order of 6.1 Gt per year (UNEP 2019). As a consequence, global and national targets for the reduction of GHG emissions and EV adoption, as well as the aspirations of the manufacturing sector, call for urgent change by 2030, with full decarbonisation by 2050. Here, we argue that a key parameter neglected in discussion of automotive decarbonisation is ‘time’. Unless the integrated timeframe for all aspects of the transition is assessed, vehicle demand and supply may not be synchronised and the goal of decarbonising the automotive sector will not be achieved by 2050.

Decarbonising the automotive sector will require significant technological innovation and social changes. These include electrification, improved fuel efficiency, fuel substitution and modal shifts such as increased use of public transport, car sharing and societal endorsement of electric vehicles. Regardless of how these changes are made, it is clear that they are all reliant on adequate, timely and sustainable supplies of raw materials.

Not only must the size of the electric vehicle fleet be increased greatly, but at the same time there is a need to develop new technologies for powertrains, batteries, battery charging, fuel cells, power electronics and vehicle bodies. While considering how these major, mainly technical, challenges can be overcome, it is also essential to assess the time required for their implementation.

## Changing raw material needs

The electrification of the automotive sector will necessitate the use of larger amounts of a wider range of metals and minerals than the internal combustion engine (ICE). The major industrial metals, such as iron, aluminium and copper, are already produced in huge quantities (tens to hundreds of millions of tonnes per annum) for myriad applications, including automotive, from mines located in many countries. In contrast, the ‘technology metals’, such as cobalt, lithium, rare earths and platinum group metals, are generally produced in much smaller amounts (hundreds to thousands of tonnes) from a small number of mines worldwide. We will require a massive and rapid increase in the production of technology metals, essential to the function and performance of electric vehicles (EVs), if we are to meet the targets of governments and the car industry.

Other materials will also be required for generating energy to charge the batteries used in EVs. The decarbonisation of automotive transport will not deliver reduced carbon emissions if the fuel used for charging is not ‘green’. Renewable energy technologies, such as photovoltaics, wind turbines and water electrolysis, rely heavily on technology metals such as rare earths, tellurium, gallium and platinum.

The demand projections for many of the minerals and metals required for decarbonisation show exponential growth up to 2050 and beyond (Hund et al. 2020). In the Stated Policies Scenario (STEP), the IEA estimates that global EV car sales will reach 15.7 million in 2025 and 27.7 million in 2030. STEP is based on the policies, regulations and targets already set by governments and industry around the world. The 2030 estimate increases significantly under the Announced Pledges Scenario (APS), which includes the most significant recent national 2030 targets as well as longer-term net zero and other pledges, to 43 million EV cars. In the Net Zero scenario, which is compatible with the Intergovernmental Panel on Climate Change assessment, which aims to limit the global temperature rise to 1.5 °C, the estimated EV car sales would reach over 65 million in 2030. To put these figures into perspective, global EV sales in 2021 were 6.6 million (IEA 2022).

**Fig. 1:**
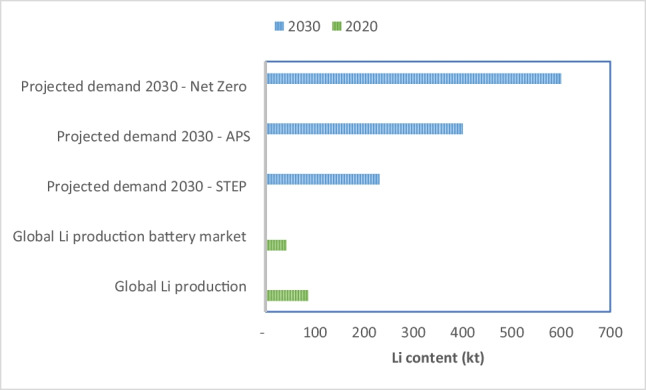
EV lithium demand in 2030 under the STEP, APS and Net Zero scenarios compared with the current (2020) global lithium production and that part of production supplied to the battery market (own calculations based on data from BGS 2021; IEA 2022)

These projections have major implications for the supply of individual battery metals. In the STEP scenario, cobalt demand for EVs will increase to nearly 63 kt/year in 2030 from 43 kt in 2020; over the same period, lithium demand will grow to about 231 kt/year from 43 kt and class I nickel^1^ to 840 kt/year from 126 kt. In the APS scenario, the growth in material demand is even greater, in many cases more than double the requirements of STEP. For lithium, there is a considerable gap between the current global lithium production and the demand projections for 2030 and an even larger gap for that part of production which is consumed by the battery market (Fig. 1). Lithium production for the battery market will have to increase rapidly in the coming decade to satisfy demand estimated in the STEP, APS and Net Zero scenarios.

In the light of these projections, it becomes critical to assess whether supply will be able to meet the forecast demand within the required timescale. It is important to emphasise that there is a clear consensus among geologists that physical availability of raw materials will not be a constraint on supply (European Commission 2011, Lusty and Gunn 2015; Worstall 2015). There have been many forecasts of the impending depletion of metals and minerals in the Earth’s crust (Cohen 2007, Giurco 2009). However, these are fundamentally flawed because they fail to understand that mineral reserves are dynamic economic entities that are continually replenished by exploration according to market needs. Published mineral reserve data should be regarded as a working inventory of what is currently available to mine and does not represent all that exists in the crust.

Although geological availability is not an issue, there are many factors that affect the accessibility of a mineral resource and may, therefore, contribute to increasing the risk of supply disruption or delay for an individual material required by the automotive sector. The importance of each varies between commodities and key-producing countries but they commonly include the following: production concentration; protectionist tendencies, such as resource nationalism, geopolitics and trade wars; the political and economic conditions in producing countries; investment availability for new mining projects; lack of exploration and underpinning geoscience research; competing demand; the by-product status of many technology metals; environmental performance and regulation in producing countries; and the social acceptability of extraction and processing activities. Less common events such as natural disasters, accidents and the COVID-19 pandemic can also have serious impacts on raw material supply. Consequently, it is particularly challenging to build robust and resilient supply chains for the automotive sector over a short timescale.

Most of these influences are dynamic in character and significant change can occur at any time. In addition, resolving such issues is time-consuming and can adversely impact the timeline of mineral projects in development. The long lead times associated with mining projects have been discussed by several authors in the past (Khan et al. 2016, Schodde 2019; Wellmer and Dalheimer 2012; Wellmer and Berner 1997). However, the implications of the long lead time for new mining alongside the electrification transition, although referenced by others (IEA 2021; Kettle 2021), have not been considered in an holistic manner that considers all tiers of the supply chain, from mineral exploration through to EV manufacture. A notable exception is the work by Heijlen et al. (2021), where they investigated the mine development pipeline (exploration and mine development stages in tier 4) for the battery metals nickel and cobalt.

## Transforming automotive supply chains

**Fig. 2:**
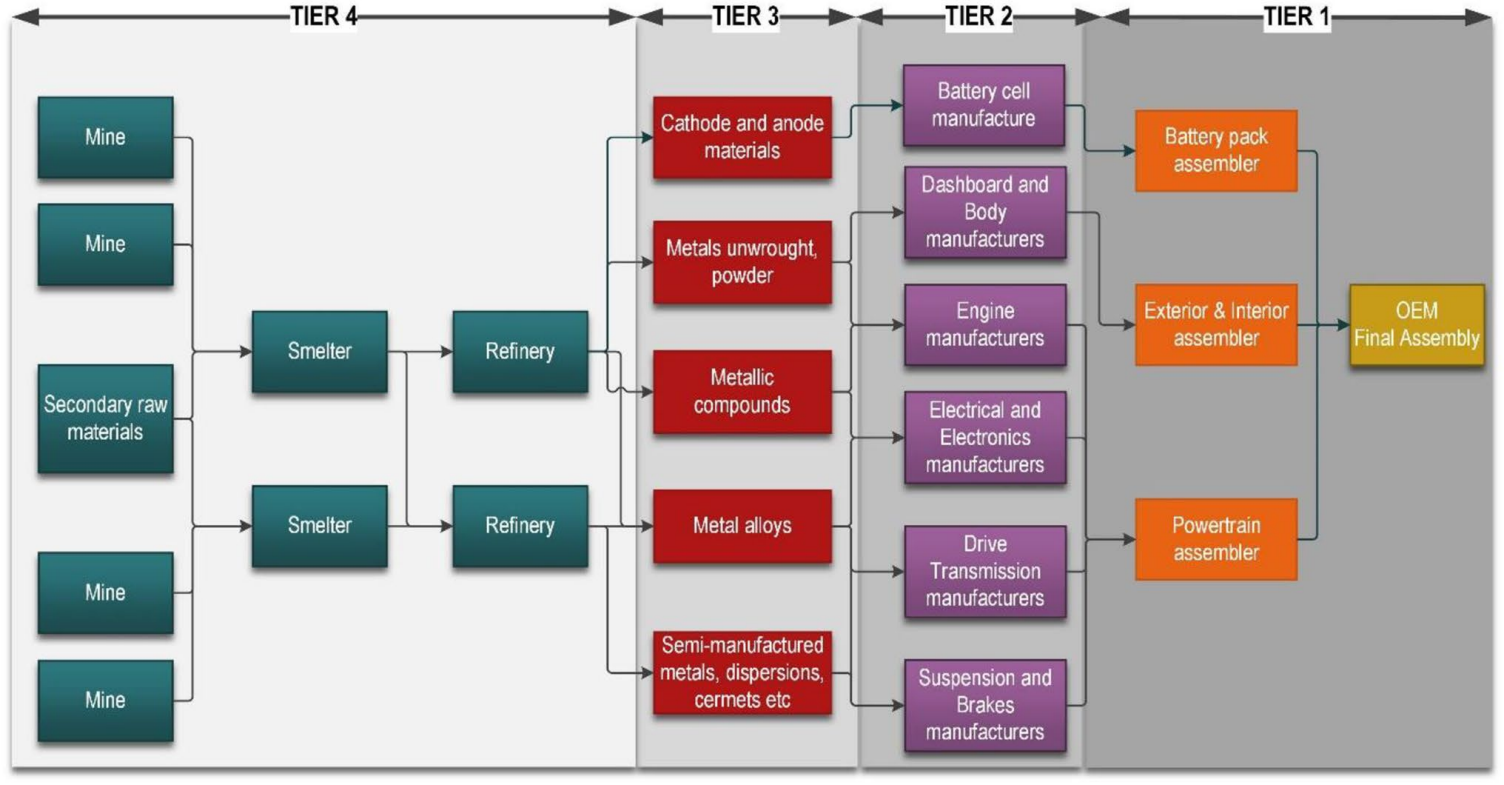
Schematic structure of the automotive supply chain (modified from (Chandra and Kamrani 2004))

Product manufacture in the automotive sector relies on complex global supply chains involving many specialist companies providing materials, components and services (Fig. 2). These supply chains are typically multi-tiered and highly dispersed in structure. The length and complexity of the supply chains may provide various benefits in term of efficiency, performance, cost, resilience and responsiveness, but may also be linked to negative environmental and social impacts.

The automotive supply chain is built around the needs of the OEMs (original equipment manufacturers). Tier 1 suppliers are companies that supply parts or systems tailored to the OEMs, while tier 2 suppliers make specialised products for OEMs across a range of sectors. For example, companies such as Intel, which produces computer chips, supply products to tier 1 automotive suppliers, but also to electrical and electronic equipment manufacturers, telecommunication industries and many more. Tier 3 suppliers are those that supply materials, such as metals, composites and glass, to a wide market rather than a single sector. The automotive sector is an important client for many tier 3 suppliers so many of their products, such as alloys and composites, are produced to comply with specific vehicle design requirements (Chandra and Kamrani 2004; Günther et al. 2015).

The supply chain does not, however, stop at tier 3. It is also essential to consider tier 4 suppliers who provide the vast range of minerals, metals and ‘intermediate’ materials used in tier 3. Tier 4 comprises a complex, dynamic global supply chain in its own right. It includes the exploration, mining and refining industries that traditionally have few direct links with OEMs. Any disruption at the tier 4 level can have serious impacts on the rest of the automotive supply chain and on delivery of the final manufactured products.

Just-in-time manufacturing (JIT), which is widely used in the automotive sector, provides substantial cost savings to OEMs by eliminating the need for large stock, warehouses and long inventories. However, it is heavily reliant on the supply chain to deliver the products needed at the right time. The timely and secure supply of materials and components is therefore fundamental to the automotive sector. Factors potentially disrupting raw material supply, such as commodity price volatility, regulatory changes affecting mine permitting and international trade policy, may have serious negative impacts on the entire production process (Coffin and Horowitz 2018; IEA 2021).

Although the electrification of the automotive sector will not, in principle, alter the multi-tiered structure of the supply chains, major changes in the technologies utilised will engender shifts in the material requirements. Some of the most sweeping changes are due to the move from ICE to electric motors and lithium-ion batteries (LIBs). Similar radical revision will take place as the complex transmission systems utilised in ICE vehicles are replaced by simpler electronic drive systems used by EVs. Some suppliers, such as engine manufacturers, will have to adapt to the new technologies or face elimination. The supply chain will need to be modified to accommodate completely new products, notably EV batteries. Without the timely installation of adequate capacity for the production of motors and batteries, the electrification of the automotive sector will be delayed.

This transformation will inevitably involve changes in the location of different parts of the supply chain. The vertical integration seen in China, from raw material production to battery and EV manufacture, as well as the increase in battery production capacity in central Europe, the USA and south-east Asia, are already leading to significant reconfiguration. Many factors may influence how and where these transformations take place and the time taken to effect such changes. These include, for example, distribution networks, trade rules, the political stability of new participants and their ability to access materials and maintain appropriate environmental standards.

However, the transformation required in tier 4 is likely to be the most problematic one. The market for battery metals has been expanding fast and, based on demand projections, the growth rate is likely to peak between 2025 and 2030. After that time, the market is projected to grow at a steady rate (Gregoir and van Acker 2022; IEA 2021). Timewise, all tiers had about 10 years (2020 to 2030) to make this transformation. Although this timeline may be adequate for tiers 1 to 3, it is generally not so for tier 4, which requires much longer lead times. Therefore, bottlenecks with supply in tier 4 could delay the development of the electrified automotive supply chain. The issues related to lead times in tier 4 are discussed in more detail in the following sections.

## From raw materials to electric vehicles—an integrated supply chain timeline

The key concern of this paper is the impact that raw material supply may have on the timely delivery of EVs to decarbonise the automotive sector. We present the different stages of the tier 4 supply chain and how they link to tier 1 to 3 by producing an integrated timeline (Fig. 4; Table 1). The component stages of each tier of the supply chain are numbered sequentially:

1. Exploration: area selection and reconnaissance surveys
2. Exploration: detailed surveys and target evaluation
3. Exploration: detailed feasibility studies
4. Mine development: permitting and regulatory compliance
5. Mine development: construction
6. Mine development: commissioning
7. Smelting and refining: development of new capacity for specific materials
8. Tier 1–3: EV design
9. Tier 1–3: development of manufacturing supply chain
10. Tier 1–3: EV production

**Table 1:** Summary of tier 4 (stages 1 to 7) indicating average duration and possible mitigation to shorten the length of each stage

Exploration
**Stage 1:** Area selection and reconnaissance surveys over large areas of hundreds or thousands of km^2^ **Average duration:** 2 to 3 years Possibility to shorten time*: 1 **Appropriate mitigation:** Availability of high-quality digital regional baseline datasets (geological, geophysical, geochemical and topographic) as well as data from previous exploration or mining activities in the license area **References:** (Gandhi and Sarkar 2016, Macheyeki, Li et al. 2020, Schodde 2019)
**Stage 2:** Detailed surveys and target evaluation of the most prospective zones (typically a few km^2^ or tens of km^2^) **Average duration:** 4 to 5 years Possibility to shorten time*: 2 **Appropriate mitigation:** Availability of high-quality geological, geophysical and geochemical data and records of previous drilling, exploration and mining. Availability of modern equipment and trained personnel for surveying, sampling, drilling and data interpretation **References:** (Schodde 2019) and authors’ own estimate
**Stage 3:** Detailed feasibility studies **Average duration:** 2 to 3 years Possibility to shorten time*: 3 **Appropriate mitigation:** Detailed deposit evaluation is time consuming and expensive. It involves various activities to determine the extent, form and quality of the deposit, such as drilling, 3D modelling and preliminary metallurgical testing **References:** (Schodde 2019) and authors’ own estimate
**Mine development**
**Stage 4:** Permitting and regulatory compliance—acquisition of mining license and environmental permits **Average duration:** 2 to 3 years Possibility to shorten time*: 1 **Appropriate mitigation:** Streamlining of the permitting process **References:** (Green and Jackson 2016; Minerals Make Life 2022; SNL Metals and Mining 2015)
**Stage 5:** Construction -procurement of equipment, construction of the mine and ancillary infrastructure **Average duration:** 5 to 7 years Possibility to shorten time*: 2 **Appropriate mitigation:** Shortening this stage is facilitated for projects in areas with active mining where infrastructure and expertise are already available **References:** (Extractives Hub 2021, Khan et al. 2016)
**Stage 6:** Commissioning—testing and optimising processes **Average duration:** c. 2 years Possibility to shorten time*: 2 **Appropriate mitigation:** Pilot-scale processing and metallurgical testing (Stage 3) could reduce risks associated with commissioning, but it is difficult to shorten this stage to less than 2 years **References:** (Extractives Hub 2021) and authors’ own estimate
**Smelting and refining**
**Stage 7:** Development of new refining capacity for specific materials **Average duration:** 5 + years Possibility to shorten time*: 1 **Appropriate mitigation:** Technology and innovation in metallurgical processing, long-term contracts with supply chain (raw materials provision and downstream supply chain) to de-risk investment; streamlined permitting systems and access to green energy sources **References:** Authors’ own estimate

*Indicative qualitative ranking of the possibility to reduce duration: (1) ample scope to reduce the time; (2) limited opportunities; (3) generally difficult.

These activities are seldom carried out in a wholly sequentially manner. More often than not, according to local circumstances, there will be an overlap of some stages leading to potential time saving. For example, in some jurisdictions when a new mine is being planned, it is mandatory to acquire certain operating permits before completion of a full bankable feasibility study. It is also important to note that the status of individual projects varies considerably. For example, a relatively advanced project at or near an existing mine will generally be able to contribute to supply more quickly than opening a new mine where previously none existed. Regardless, the existence of adequate mineral resources that can be mined, processed and refined in an efficient and sustainable manner is a fundamental requirement to meet the forecast increased demand for the minerals and metals needed for use in EVs.

### Exploration

The first stage in the material supply chain comprises exploration for the minerals and metals needed for the automotive sector. Exploration is the process by which mineral resources (potentially minable bodies of rock or mineral) are identified and then converted to mineral reserves (material which can currently be mined technically, legally and economically). Without exploration, there can be no reserves and consequently no mining.

Exploration is carried out in a series of stages which serve to identify a minable deposit. Each successive stage requires increasingly detailed and expensive investigations. Throughout exploration and mine development technical, funding and market risks persist to varying degrees, while continuing political risk can, in an instant, undermine the viability of a project. This risk varies greatly between countries and is largely dependent on the prevailing political stability and governance standards within the country in which the deposit is located (Eggert 2010, Trench et al. 2014).

The first stage of the exploration cycle comprises the selection of an area that is considered favourable for the occurrence of a particular type of deposit which may contain the target metal or mineral in potentially economic amounts (stage 1). This selection is based on an evaluation of the available geoscience data in the light of accumulated knowledge and experience of seeking similar deposits elsewhere. Initial reconnaissance exploration, typically over large areas of hundreds or thousands of square kilometres, seeks to identify the ‘fingerprint’ of a potential deposit and thus locate promising targets. The initial area selection is based on many factors, such as the presence of known mineral occurrences and deposits, the geological setting, the age and type of rocks present, and the presence of certain geochemical and/or geophysical characteristics. Thereafter field investigations, commonly including geological mapping and geochemical and geophysical surveys, are undertaken to assess the mineral potential of the chosen area.

Our ability to explore successfully depends fundamentally on the availability of reliable deposit models that can guide where and how exploration should be carried out. For the major metals, such as iron, aluminium and copper, which have been widely used by industry for many decades, we have well-constrained models that define the deposit fingerprint that may indicate the presence of a deposit. In contrast, many technology metals have only recently been of commercial interest and the markets are very small. Consequently, for these materials, our deposit models are poorly constrained and do not provide reliable guidance on where resources might be found. In order to improve exploration targeting and effectiveness, it is, therefore, important that we improve our knowledge of how and where these materials are concentrated in the Earth’s crust.

When we have a reasonable understanding of how a deposit is formed, we can look for evidence of these processes in available datasets, such as geological maps and other regional survey data. However, there is considerable variation in the availability of this fundamental data which underpins mineral exploration. For example, a wide range of data and materials specific to critical minerals is available in Australia (Geoscience Australia 2022). In contrast, there are few modern geological maps available for many countries, notably in sub-Saharan Africa and Latin America.

Given encouraging results at the reconnaissance stage, more detailed exploration (stage 2) is carried out over the most prospective zones that are typically a few km^2^ or tens of km^2^ in size. This stage generally comprises geological, geophysical and geochemical surveys, sometimes followed by shallow drilling.

If the results of the preliminary target appraisal are positive then, given the availability of funding, the next stage involves more detailed deposit evaluation to determine the extent and quality of the mineralisation below the ground surface. This is accomplished through drilling to provide a three-dimensional picture of the depth, form, extent, grade and continuity of the mineralised body. Some preliminary metallurgical testing is often undertaken at this stage to evaluate if the target metal can be effectively extracted and separated from its host rock.

It may take many years from initial conceptual development and area selection to completion of the reconnaissance and detailed exploration stages. The actual timescale is dependent on numerous variables such as the target commodity, the type and location of the deposit, market conditions and the availability of funds, the host government’s policies and permitting procedures, and the relationship with local communities. Few exploration projects actually proceed beyond the stage of detailed exploration. The reasons for this are many and varied, commonly involving some combination of economic, technical, environmental, social or legal issues. Some projects may be completely abandoned, while others may be temporarily halted for periods that may last from a few months up to many years, until, for example, market conditions improve, a new technology for mining or ore processing is developed or the host government changes a policy which had previously been a barrier to re-opening. What is clear is that in the majority of cases, providing new metal supply is quite unlike turning on a tap; bringing a new mine into production or re-opening an old one is seldom straightforward and rarely accomplished without delay at some stage.

There are numerous examples from every continent that serve to illustrate the time taken from deposit discovery to the opening of a new mine (Schodde 2019). A recent study assessing 100 gold and copper discoveries worldwide found that on average, it took 12 years to make the discovery and that over the last 40 years, the rate of discovery appears to be slowing down (Khan et al. 2016, Schodde 2019). There is little comparable data for the technology metals although a recent analysis of historical nickel mine development showed that the lead time from the start of exploration to the beginning of mine production increased from 8 to 12 years between 2000 and 2020 (Heijlen et al. 2021).

In a few cases, given favourable economic, political and social conditions, projects are taken to the next stage which is a full bankable feasibility study (stage 3). This involves more comprehensive technical investigations to confirm the size and grade of the resource, to determine how the ore can be mined and processed and the valuable metal or mineral extracted. The construction of a pilot plant to optimise the mineral separation and recovery of the target material is sometimes included at this stage. At the same time, a full financial analysis is undertaken to calculate development and operational costs and to confirm economic viability. This feasibility study, which must conform to an agreed international standard, forms the basis for attracting further investment to fund mine development, either through a public listing on a stock exchange or from private investors. It is also required in most countries in order to obtain the permission of the host government to mine.

Although every case is different, it is possible to provide some general estimates of the time taken for each exploration stage. These are summarised in Fig. 4: regional selection and reconnaissance exploration typically takes 4–5 years, while subsequent detailed surveys and target evaluation commonly last 2–3 years. The detailed investigations required for a feasibility study are technically complex and generally take 2–3 years to complete. Although the duration of each stage is subject to considerable variation dependent on local conditions and the availability of funds, the total time for completion of the exploration phase is commonly 8–11 years.

### Mine development

The mine development stage starts with the acquisition of a mining licence (stage 4), which involves a commitment by the project owner to undertake work in compliance with a raft of technical, environmental, fiscal and administrative legislation. Following payment of the appropriate fees, the host government grants permission for the specified activities to be undertaken over an agreed timeframe.

Stage 5 begins with design of the mine, which is followed by a range of engineering, procurement and construction processes. This is followed by procurement of the necessary equipment and materials and securing the services of contractors to undertake the construction. The work begins with preparation of the site for erecting mine plant and for installation of the required infrastructure needed for the mine operations. For an open-pit mine, preparatory work includes clearing vegetation, pre-stripping, removal of overburden material and stockpiling of any excavated ore for later processing when a plant is in place. In the case of an underground mine, access to the ore involves the excavation of shafts and ramps and the preparation of the orebody for first production (Extractives Hub 2021). The process of procuring equipment with a long lead time, such as crushers, trucks and drilling equipment, is initiated early in stage 5 (Fig. 4). Other activities undertaken at this time include the construction and installation of infrastructure (roads, drainage, power, transportation and telecommunications), processing plants and supporting facilities for the work force (Extractives Hub 2021).

Commissioning of the mine, stage 6, takes place towards the end of construction. This phase includes the testing of various installations, such as processing plants, to ensure that performance aligns with earlier metallurgical testing and pilot-scale production undertaken during stage 3. It is during commissioning that the scale-up of production is tested to its full extent. This commonly entails a range of adjustments to equipment and operational parameters to optimise performance. At this stage, the training of personnel is also completed to ensure efficient and safe operation of all aspects of the mining operation (Extractives Hub 2021).

Mine development typically takes between 9 and 12 years overall, depending on the location of the resource, its accessibility, size and grade, the financial circumstances of the project, country-related factors (e.g. economic and political stability, governance, regulatory framework) and the prevailing commodity prices. The acquisition of a mining licence typically takes 2 to 3 years depending on the location of the deposit and the permitting procedures of the host country (stage 4) (Khan et al. 2016). Mine construction (stage 5) commonly takes between 5 and 7 years, with commissioning (stage 6) adding an average about 2 years to the timeframe.

Mine development is highly capital intensive and developers will only commit to it once all necessary approvals and feasibility studies are in place and adequate investment is secured. It is also in their interest to minimise the mine construction time to ensure not only rapid payback of the investment made, but also to minimise the risk of the conclusions of previous studies becoming outdated and thus invalid (Extractives Hub 2021; Gajigo et al. 2012; Khan et al. 2016).

### Global exploration, mine development and case studies

Global mineral exploration remains strongly focussed on gold and copper as it has for many years (Cobalt Institute 2022, S&P Global 2022). However, recently there has been a considerable increase in activity related to ‘technology’ metals, such as lithium and cobalt. The ‘major’ integrated, multi-commodity mining companies have also become increasingly involved in these commodities in what was previously a sector dominated by ‘junior’ companies (S&P Global 2022).

**Fig. 3:**
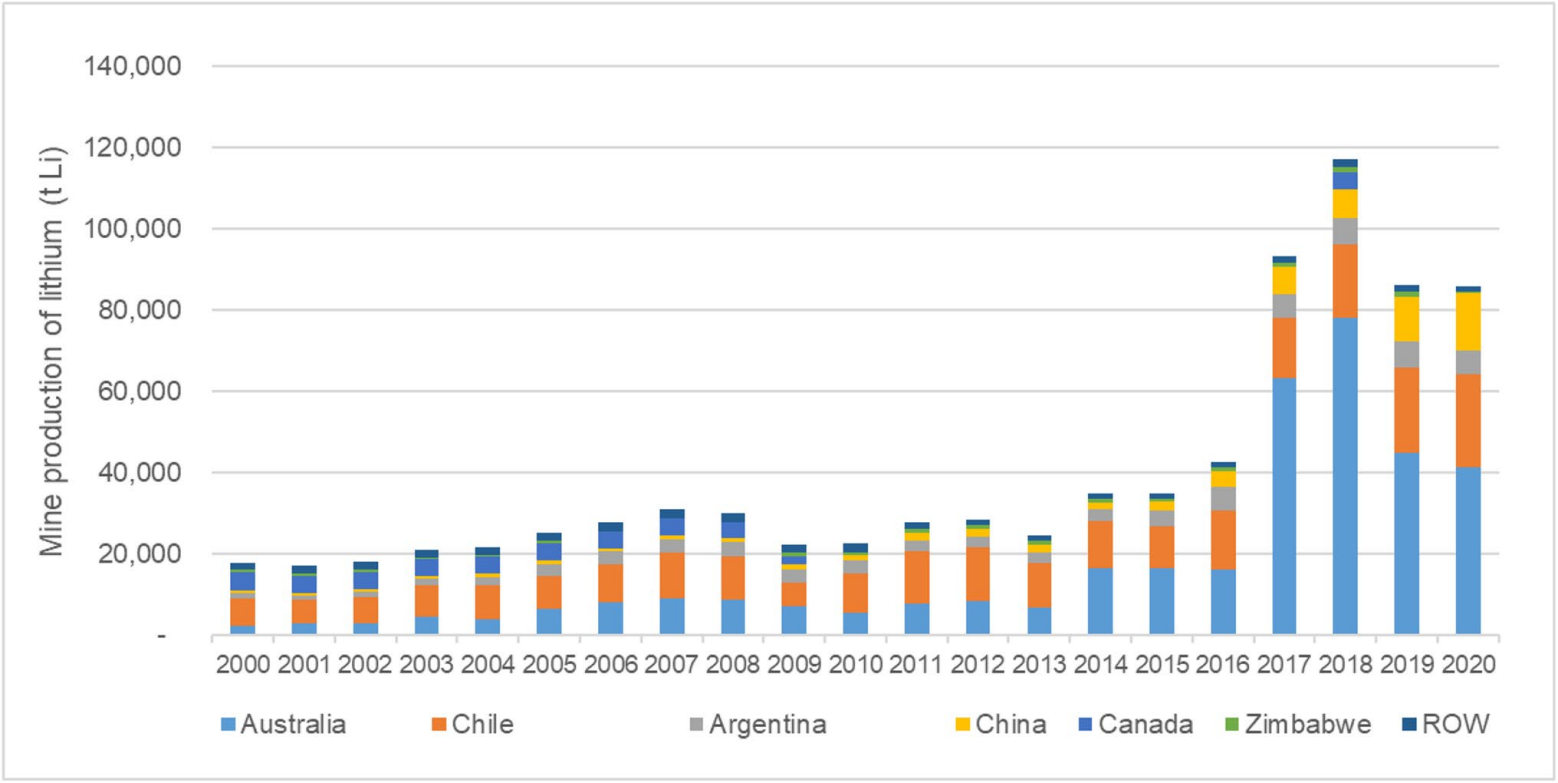
The distribution of global lithium production, 2000–2020 (data from BGS World Mineral Statistics database)

Lithium deposits and occurrences of several types are distributed widely across the world (Shaw 2021). In addition to established extraction in Australia, Chile, Argentina and China (Fig. 3), many advanced projects are being evaluated in these countries and elsewhere, such as in the USA and Canada. Furthermore, on the basis of historic exploration and mining activity, good geological potential for lithium has been widely demonstrated, notably in several African countries such as Zimbabwe, Namibia, Ghana, the Democratic Republic of Congo (DRC) and Mali (Goodenough et al. 2021).

There is also considerable interest in diversifying the supply base for cobalt and thus reducing the current reliance on the DRC, which accounted for more than 74% of global mine production in 2021 (Cobalt Institute 2022). Advanced cobalt projects are being evaluated on every continent (Petavratzi 2019), with particular emphasis on locations where cobalt resources are already known and, in some cases, previously mined. Examples include the Idaho Cobalt Operations (ICO) of Jervois in ID, USA (Jervois 2022), and the NICO deposit in the Northwest Territories of Canada (Fortune Minerals Ltd. 2019). In Europe, Horn et al. (2020) identified cobalt resources in a range of geological settings at numerous locations. The greatest cobalt resource potential lies in laterite deposits in the Balkans and Turkey, and in magmatic and black-shale-hosted deposits in Fennoscandia (Horn et al. 2020).

There is also considerable global exploration underway for nickel. In the next few years, new mine supply is expected to be dominated by greenfield and brownfield projects under development in Indonesia. Some of these operations will also recover cobalt from the laterite ores. Additional nickel production is also expected in Australia, Brazil and Canada.

The variation in the timescales for exploration and mine development between countries is well illustrated by lithium in Chile and Australia. Both countries have been producing lithium since the beginning of the millennium, although it was only in 2014 that there became a high level of global awareness concerning increased future demand for lithium. While both countries have plans for increased production from their existing reserves, Chilean lithium companies have only raised production marginally (Fig. 3), and no permission for new mines has been granted since 2014 (Sherwood 2019). Australian projects, on the other hand, have managed to increase extraction considerably from existing projects (Greenbushes, Wodgina, Mount Marion, Mount Cattlin) and by opening new mines (Pilgangoora and Bald Hill). This has led to a fivefold increase in lithium production in Australia within a period of 5 years (Champion 2018).

In the case of Chile, it is not a shortage of reserves that has restricted mine development, but a range of other factors including the following: the lack of fair and transparent implementation of the regulatory and permitting framework; the absence of adequate baseline hydrogeological data at the watershed-scale that can underpin new mining concessions, environmental assessment and management; the lack of coordination among government, industry and local community stakeholders; ongoing socio-environmental conflicts; and the shortage of secure investment (Obaya and Pascuini 2020). In addition, the use of solar evaporation for concentrating lithium in brines creates long lead times to production as a range of technical, geographical and climatic challenges have to be addressed first (Hocking et al. 2016). In marked contrast, a definitive feasibility study on the major Pilgangoora lithium-tantalum project in Western Australia was completed in 2016. Mine construction started in 2017 and the first shipment of lithium concentrate was exported in 2018 (Pilbara Minerals 2018). This is the fastest major lithium development in Australia in recent years. The key factors to its rapid development were binding offtake agreements with leading global partners from the downstream supply chain and the presence of an efficient regulatory and permitting framework in Western Australia. Although the timeline of this project is quite unusual, it highlights that convergence across the supply chain, adequate investment and good mineral governance can dramatically reduce the lead time for a new mine project. Ultimately, however, every deposit is different and the factors that may influence project development are many and varied.

The Norra Kärr rare earth element (REE) project in southern Sweden provides another pertinent example. This deposit, discovered in 2009, contains major resources of the much sought-after heavy rare earth elements (Leading Edge 2021a). A 25-year mining licence was granted in 2013 but this led to large-scale opposition and massive protests related chiefly to environmental concerns. In 2016, the extraction licence was overturned by the Supreme Administrative Court of Sweden and in May 2021 the Mining Inspectorate of Sweden rejected the mining lease application made by Leading Edge Materials, the owners of the deposit. The company subsequently appealed this decision (Leading Edge 2021b), although more recently this has been retracted and a Natura 2000 permit application for the project has been initiated (Leading Edge 2022).

The Sotkamo project in eastern Finland provides another illustration of the challenges involved in opening a new mine. The deposit at Sotkamo (formerly known as Talvivaara) was discovered in 1977, but its low grade meant that it was not economic to mine at that time (Sairinen et al. 2017). However, with the advent of bioheap leaching technology, commercial operation started in 2008 with nickel and zinc as the main products. However, as early as 2009, operations were beset by a raft of environmental problems leading to serious concerns at both the local and national scales. Despite repeated attempts to solve these problems, the owner of the mine went bankrupt in 2014. A government-owned company, Terrafame, took over the operations in the following year since when the mining and environmental performance have greatly improved. The mine is currently a major producer of nickel and zinc, together with by-product cobalt and copper. Terrafame also received approval to produce battery-grade nickel and cobalt chemicals from April 2021 thus creating an integrated supply chain from mine to battery chemicals at one site (Terrafame 2021a).

### Smelting and refining

Extractive metallurgy, which is an integral part of tier 4, comprises the conversion of raw minerals produced by mining into purified metals or compounds. Extractive metallurgy, here referred to as smelting and refining (stage 7), involves the use of various technologies that may be used on their own or in combination to separate the metals from their host ores and to make the products required for particular applications. Most extractive metallurgical processes used today belong to three major classes: pyrometallurgy, also referred to as smelting, which relies on reactions at high temperatures between gases and solids, or gases and molten materials; hydrometallurgy, which uses aqueous solutions to extract metals from ores; and electrometallurgy, which utilises electrochemical processes, such as electrowinning. In certain cases, for example for lithium recovery from brines, the extractive metallurgy includes chemical processes taking place through solar evaporation that enrich the brine in lithium.

It is important to note two key points regarding the extractive metallurgy routes which are utilised. First, each metal extraction flowsheet is unique to a particular deposit or ore type, and, secondly, that no two metals are recovered using exactly the same sequence of extraction steps. Numerous factors influence the extractive metallurgy process routes that are adopted. These include variables such as the mineralogy of the feedstock (e.g. oxide, sulphide, silicate and other forms), the physical and chemical characteristics of the ore body and associations with other constituents, the thermodynamic reactivity of the target metals and their melting and boiling points (Rankin 2011). Another consideration, which is particularly important for some technology metals, such as cobalt, that are produced as by-products of the extraction of major industrial metals like copper and nickel, is whether extraction is intended to recover only a single metal with others being effectively lost and discarded as waste.

The increasing demand for metals such as lithium, cobalt, nickel and manganese used in batteries, but also rare earths for electric powertrains, will require major scaling up of not just mine production, but also of smelting and refining. Although some refining capacity is already in place, this is not considered sufficient to satisfy the projected demand or the requirements for specific intermediate compounds (Gregoir and van Acker 2022). The ‘battery grade’ label that we now see associated with battery raw materials, such as lithium, cobalt and nickel, requires high purity compounds, such as battery grade lithium carbonate or lithium hydroxide monohydrate, battery grade cobalt sulphate and class 1 battery grade nickel sulphate. Processing and refining operations for all battery raw materials are concentrated in a few countries. For example, China has about 40% of the global refining capacity for copper, 35% for nickel, 60% for lithium, 65% for cobalt and 90% for rare earths. Refining capacity also exists in other places, such as in Chile and Argentina for lithium, Indonesia and Japan for nickel, Finland and Belgium for cobalt, Chile and Japan for copper and Malaysia and Estonia for rare earths (IEA 2021). New processing facilities have also been announced. For example, in Western Australia, the Kwinana lithium has been commissioned and started production of lithium hydroxide (battery grade) in 2022 (Tianqui Lithium Energy Australia 2022). The Kemerton lithium refinery, also in Western Australia, is being constructed to produce lithium hydroxide (Albermarle 2022; Department of Industry Science and Resources 2021). The POSCO nickel sulphate refining facility in South Korea (Jung-hwan Hwang 2021). Other planned developments include the expansion of the Nornickel Harjavalta nickel refinery in Finland and several high-pressure acid leaching plants in Indonesia aiming to produce a mixed nickel–cobalt hydroxide precipitate (Cobalt Institute 2022). However, the capacity of existing and planned refining facilities to provide the required speciality grades is unlikely to be adequate to meet the projected demand.

Numerous other factors need to be considered when developing additional extractive metallurgy capacity. These include the environmental footprint of such operations, energy and water requirements and access to infrastructure for transporting feedstock and bulk process chemicals. Other important considerations that can influence the pace of new development include the lack of standardisation for battery grade materials, the availability of investment and the requirements of OEMs and consumers for transparency and traceability.

**Fig. 4:**
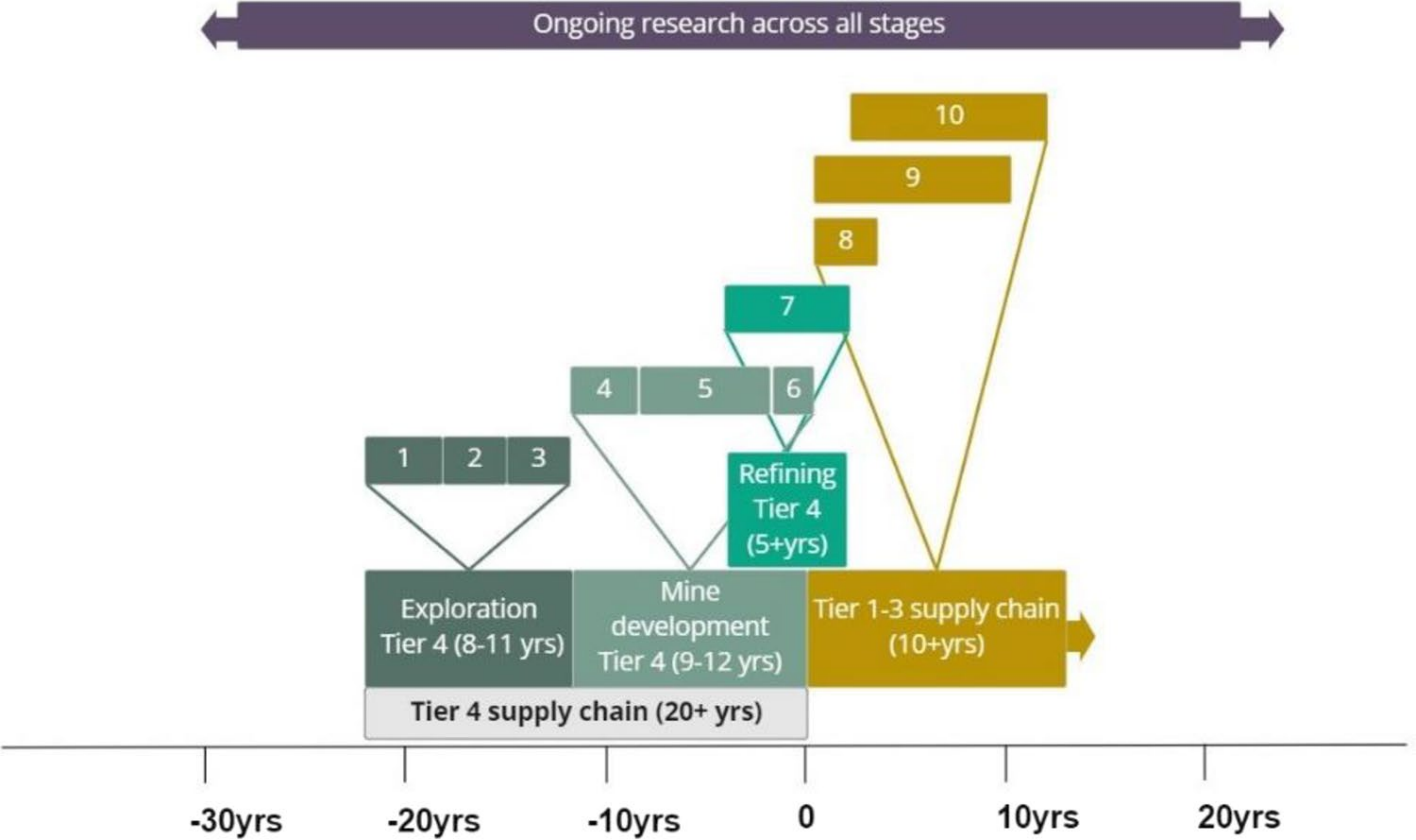
Indicative timeline for the EV supply chain. Stages 1 to 10 are summarised in Table 1. There is also a continuing need for research at all stages, including geoscience, mining, metallurgy and manufacturing

In principle, for mine development to proceed, some refining capacity needs to be in place to ensure the material journey is continuous. Accordingly, new refining capacity is developed in parallel with mine development and the tier 1–3 supply chain (Fig. 4). It is estimated to take an average of 5 years for new refinery production to come on stream.

Continued research is required in the extractive metallurgy field to ensure that materials of the right grade and form reach the market, and also to minimise many of the key challenges associated with smelting and refining, such as the reduction of environmental impacts, improved traceability and the establishment of circular economy procedures for material recovery.

### Tier 1–3 development

The automotive sector is undergoing fundamental technological disruption that influences business models and supply chains. The timescale of this transformation is driven by forecast EV demand models, CO_2_ emission targets for passenger cars and government policies for EV deployment. The lead time that the automotive sector requires to deliver the EV vehicles of 2030 is estimated to be approximately 10 years (stages 8 to 10–3). The OEMs are, therefore, currently developing the capacity to deliver their plans for the early 2030s. The EV design and development stages (stage 8), which have already started, take between 3 and 4 years with mass production of electric vehicles following afterwards (Faraday Challenge 2018). Within this 10-year timeframe, many new functions have to be developed and integrated to operate in an efficient and coordinated manner to meet EV demand, including the development of the individual parts of tier 1 to 3 supply chain (stage 9). These include manufacture of electrodes (cathodes and anodes), cells and battery packs, in addition to the other components shown in Fig. 3. Additional key requirements include the development of new powertrain manufacturing capacity and associated supply chains for components needed in new engines, as well as a trained and skilled workforce. The EV production stage (stage 10) is estimated to require a lead time of 10 years (Faraday Challenge 2018). This happens in parallel with the supply chain development (stage 9), which is continually modified and strengthened as the EV market grows. Stages 9 and 10 continue into the future as mass adoption of electrification in transport becomes the norm. However, they are likely to have to change to accommodate new technologies, such as hydrogen-powered cars and autonomous vehicles.

Research focussed on stages 8 to 10 is being undertaken by both governments and companies. Key research topics include the manufacture and performance of battery packs, the design of vehicles and electric powertrains, production optimisation processes, the assessment of the life cycle impacts of products and the development of circular economy strategies for end-of-life EVs.

## Discussion

### Challenges to the development of new projects

Our review of the integrated automotive timeline clearly demonstrates the long lead times, often exceeding 30 years, to bring raw materials from a new mine into EV production. Tier 4 includes the stages of longest duration which together commonly exceed 20 years. Consequently, to ensure adequate raw material supplies for the anticipated ramp-up in EV production, there should be new mines in the pipeline now, based on discoveries made some decades ago. However, we are not currently in this position; rather, many new projects are in the early stages of exploration and their future is not assured due to continuing market, societal, environmental and political uncertainties for most technology metals needed by the EV industry. Increased production in recent years has mostly been achieved by a favourable combination of local circumstances and economic conditions. Most increases are linked to existing projects that have managed to secure investment to upscale their operations or from opportunistic acts following recognition that some of these materials, which previously had limited value, could become valuable by-products of operations extracting other metals. For example, the Sotkamo nickel–cobalt operation in Finland (Terrafame 2021b), the Metalkol RTR cobalt-copper tailings project in DRC (ERG 2021) and the Pilgangoora lithium mine in Australia (Mining Technology 2021a) largely owe their current status to the recent upswing in demand for battery raw materials. At the same time, the increased involvement of downstream companies in mining projects has also contributed to the early advancement of some projects. For example, BMW has announced its planned involvement in lithium extraction in Argentina and in cobalt mining in Morocco (BMW Group 2021). These investments will contribute to accelerated supply of these metals for use in EV batteries.

Projects aiming at developing new mines and refineries are exposed to a wide range of vulnerabilities, both technical and non-technical (Eggert 2010, Trench et al. 2014). Technical risks include those associated with uncertainties in geology, engineering, extractive metallurgy, operational safety, environmental impacts and the availability of materials and infrastructure (power, water and transport). Non-technical risks are many and varied. They include the following: the stability of government policy and regulation in the host jurisdiction; uncertainties in commodity markets and funding availability; social acceptance by affected parties; the requirement for transparency and traceability across the supply chain; risk to corporate reputation (e.g. on brand protection within the automotive sector); unregulated artisanal mining activities; and availability of a skilled workforce.

The financial investment associated with progressing a project from the first stage of exploration through to completion of a definitive bankable feasibility study is substantial and increases at each stage (Eggert, 2010). Today, the construction and commissioning of a new mine typically costs hundreds of millions of dollars. In recent decades, most new ‘greenfield’ discoveries, namely those in areas lacking any previous mining, have been made by small, so-called junior, exploration companies who undertake initial reconnaissance exploration and then generally sell on to, or seek partnership with, larger companies who have the financial muscle to advance the project. However, a key issue for greenfield projects, in contrast with development around an existing mine, is that they do not have current production to provide income and assurance to investors. History also shows that the probability of such exploration projects becoming producing mines is very low. For example, there was a surge in exploration for the REE in the 2-year period following the 2010 trade dispute between Japan and China over rare earth supplies. Although more than 400 exploration projects were active at that time, only two REE mines have opened outside China since (Machacek and Kalvig 2016; Van Gosen et al. 2014). Financial risk for technology metals is also increased because their markets are small, relatively immature and lacking in transparency with no publicly quoted prices for many of them. Together these factors combine to deter investment in new projects focussed on these metals.

The legacy of poor environmental performance of past mining, together with our aspiration to develop new projects that follow sustainability principles, means that environmental issues have to be considered and measured throughout tier 4, from the exploration phase onwards. Regulatory requirements, licensing processes and the ever-increasing need for life-cycle thinking in assessing the environmental impacts of mining projects require acquisition of more data, followed by modelling and in-depth analysis. The cost and duration of new project development are inevitably increased as a result.

Ensuring that projects have a social licence to operate has also become increasingly important in recent years and is now a prerequisite for the successful development of a new mining project. In order to obtain this licence, full and transparent engagement with communities is required from the start of a project. This helps to build an environment of trust and mutual respect while ensuring that all parties are fully informed, their rights respected and the derived benefits fairly shared throughout the life of a mine and following its closure. Although the necessity to acquire a social licence has added to both the cost and duration of new development, failure to do so has led to many project delays and cancellations in recent years. Notable examples include the following: the Norra Karr REE project in Sweden (Leading Edge 2021a), where its initial licence was revoked (the Uyuni lithium brine project in Bolivia) (Sanchez-Lopez 2020); and the Sakatti copper-nickel-platinum group metal project in Finland (Leena et al. 2019).

Market growth and transformation will require further expansion in refining capacity to meet raw material demand, to accommodate changes in battery chemistry and to handle the increasing stock of end-of-life EV batteries. Existing refining and conversion capacity is currently monopolised by a small number of industry players and nations and this is perceived as a likely bottleneck to long-term secure supply of raw materials. Technology roadmaps from the automotive sector highlight that there will be significant changes in battery chemistry, which will require adjustments in smelting and refining to ensure that the right grades and forms of materials are produced (IEA 2021). Similarly, different treatment procedures will be required to recover the cathode and anode components from end-of life-batteries. In other words, there will be a continuing requirement, beyond the estimated initial 5-year period, for significant changes to be made to the smelting and refining processes. Without the appropriate smelting and refining capacity, the planned decarbonisation of transport cannot be achieved.

The burden of increasing regulation, the greater involvement of diverse stakeholder groups and growing pressure for greater transparency and traceability in raw material supply chains combine to further raise the barriers to new mining and refining projects. This is particularly so in advanced industrialised economies where new industrial development is not always welcome and environmental concern tends to be high. For many technology metals, such as those needed for EVs, the barriers to development are even greater. Although demand for such metals is growing rapidly, they remain of limited interest to most major mining corporations and their production is commonly restricted to a few companies operating in a small number of countries.

### Improving the security and sustainability of the supply of technology metals

It might be considered that regulatory barriers and growing societal and environmental concerns present an almost insurmountable challenge to the timely supply of ever-increasing quantities of metals and minerals needed for decarbonisation. However, while there is no universal panacea and few quick fixes for scaling up production rapidly, there are many things that can be done to help achieve this goal. The measures discussed here, and summarised in Table 1, focus on tier 4 and will assist in improving the security of sustainable raw material supply but will not necessarily compress the overall timescale for delivery.

It is also important to consider the increasing expectation for the circular economy to compensate for supply bottlenecks associated with primary sources of technology metals. In principle, this is not unreasonable although our knowledge of where technology metals reside in end-of-life products is limited. In addition, it will take many years to build up sufficient stocks of end-of-life products and to develop the appropriate infrastructure and technology to close the loop for these resources. Consequently, the contribution of the circular economy to ensuring material supply to the automotive sector in the timescale considered in this study will remain limited (Bloodworth et al. 2019, Hund et al. 2020).

#### The role of governments

The technical challenges associated with providing secure, sustainable and timely supplies are best tackled through continuing programmes of multi-disciplinary, collaborative international research. Fundamental research challenges include improving our understanding of how technology metals are concentrated in the Earth’s crust, developing methods to quantify the environmental impacts of mine extraction and improving the technologies for the extraction and refining of specific metals. At the same time, governments should be working to transform the results of the academic research into policy, strategy and best-practice guidance.

We have discussed the urgent need to find more resources and to be able to mine, process and use them efficiently, safely and with minimal environmental impact. However, there remain substantial knowledge gaps which constrain our ability to meet this aspiration within a short timescale. For example, research into the formation of ore deposits is inevitably complex and time-consuming. No two metals are the same so we need to study a range of geological environments to ascertain where the greatest potential lies for the development of economic resources of each one. However, the results of such research cannot be applied unless basic geoscience data are available for the target areas. The fundamental requirement is for a modern geological map, ideally supplemented by regional geophysical and geochemical datasets, which helps to identify areas favourable for the occurrence of new deposits. The availability of such data varies greatly across the world. In some countries, there may be no or limited such data. In contrast, in some jurisdictions where mining is economically important and where governments seek to promote exploration investment, modern high-resolution digital geoscience datasets are increasingly being made available. The availability of good geoscience data is invaluable for progressing quickly to mine development.

The availability of funding is commonly a major obstacle to the development of new mining and metallurgical projects. There is no single route to mitigating financial risks as the triggers are complex and variable. Commodity markets and price volatility, the lack of supportive regulatory and governance frameworks, the perceived environmental consequences of a project, the attitudes of affected communities and uncertainties in demand projections can all lead to difficulties in raising finance. To identify appropriate mitigation, we need to assess the scale and scope of these risks. For example, risks associated with local environmental conditions, such as loss of biodiversity, land erosion and issues with waste management and air pollution, could be mitigated if appropriate governance procedures were in place. However, environmental and mineral governance vary widely across the globe and are seldom accommodated under a single framework. There is a need, therefore, for better integrated and holistic regulatory frameworks of mineral and environmental governance, which would streamline the highly bureaucratic and disconnected procedures prevailing in many jurisdictions. Social participation is an important component of mine licensing procedures, but often implemented poorly resulting in local conflicts and project delays. Improvements could be achieved through robust regulations and procedures which ensure that local communities are actively involved throughout the lifetime of a project and that they enjoy a fair share of the profits.

The presence of established drilling, mining and laboratory services and equipment manufacturers, together with the availability of skilled labour and high-quality infrastructure, can also serve to expedite project development and provide employment opportunities. Some governments offer a range of additional technical support including the provision of expert advice and web portals to access databases and publications. Some jurisdictions also carry out pre-competitive reconnaissance exploration, sometimes including drilling, to promote exploration for particular commodities in specific areas. These measures, individually or collectively, can help reduce uncertainties around new projects, boost investor confidence and ultimately shorten the timeline of project development.

Numerous diverse measures have been taken by the governments of consuming countries to mitigate the risks associated with the concentration of production of raw and refined materials in a few locations and the consequent reliance on imported supplies. The EU and the USA have been particularly active in seeking to diversify the supply base and reduce dependence on foreign suppliers. Since publishing its first list of critical raw materials in 2011 (European Commission 2011), and more recently in response to the decarbonisation agenda, the EU has been active in building resilient material supply chains and developing industrial ecosystems in Europe. Key activities have included raw material diplomacy, removing trade barriers, exploring domestic resource potential, technological innovation to reduce critical raw material (CRM) dependency, improving the knowledge of CRM supply chains and the development of industrial ecosystems (e.g. on LIBs). In 2020, the EU established the European Raw Materials Alliance (ERMA) which aims to address the challenge of securing access to sustainable raw materials, advanced materials and industrial processing know-how (European Commission 2021a). On the transport front, it established the EU Battery Alliance in 2017 which aims to develop a sustainable battery value chain in Europe. The EU Battery Alliance has its foundation in the EC’s strategic action plan for batteries, which sets out a comprehensive set of regulatory and non-regulatory measures to support all segments of the battery value chain (European Commission 2021b).

In the USA, concern about CRM started in 2008 with the publication of a report by the US National Research Council (National Research Council 2008). In 2018, the US government published a list of 35 critical raw materials for the USA (Office of the Secretary Interior 2018). This was followed by the American Mineral Security Act which aims to facilitate the ongoing monitoring of CRM supply and demand, to support domestic CRM exploration and to streamline the permitting and reporting procedures in the USA (Senate—Energy and Natural Resources 2019). It also supports the establishment of R&D on critical minerals substitution and recycling, as well as material efficiencies throughout the supply chain and the training of domestic skilled personnel across the CRM field. Following the onset of the COVID-19 pandemic, the US government undertook a rapid review of critical supply chains and made a series of recommendations for mitigating vulnerabilities that can harm the US economy (The White House 2021). The US government is also continually refining its methodology for identifying those materials important to national security and the economy which are at risk of supply disruption (Nassar et al. 2020, Nassar and Fortier 2021). It has undertaken to regularly review potentially critical raw materials, with the latest list published in February 2022 (USGS 2022).

The approaches taken by EU and the USA provide good examples of the broad-ranging strategies that nations and economic regions have put in place to alleviate issues related to the security of supply of raw materials. However, although these actions are likely to mitigate material supply risks in the long term, they will take time to implement and are unlikely to shorten the EV supply chain radically in the near term.

#### The role of industry

Beside governments, industry also has an important role to play in mitigating raw material supply risk through supply chain convergence. This involves international partnerships and collaboration across the whole supply chain. Industries from tiers that do not normally interact should collaborate closely to develop long-term plans for the supply of battery raw materials. Investments by OEMs in mining projects illustrate current supply chain convergence and the benefits accruing to all parties, especially in terms of financial security. Notable examples include investments by Tesla in raw material projects (Tier 3 and 4) in the USA (Kavanagh 2020) and Australia (Benchmark Mineral Intelligence 2021, Mining Technology 2021b). Other OEMs, such as BMW, are working closely with raw material providers in Australia and Argentina and have set up contracts to ensure secure delivery of battery metals to cell manufacturers (BMW Group 2021). Toyota has direct involvement in the lithium salar de Olaroz salt lake project in Argentina (Toyota Tsusho Corporation 2021), and Gangfeng is investing in the Cauchari–Olaroz project (Lithium Americas 2019). Others, such as Audi, are exploring the potential of sourcing battery metals from the circular economy through collaboration with UMICORE to develop a closed-loop system for battery recycling (Umicore 2018).

#### Collaboration between government and industry

Joint actions by government and industry can also expedite the EV transition, ensuring supply chain resilience and reducing the timescale. China has been particularly successful in this regard by developing industrial strategies that account for raw material requirements and promote the exploitation of domestic resources, the development of conversion capacity and integrated supply chains. These have all been facilitated by public–private partnerships and direct investment by China overseas. For example, the budget for policies related to new energy vehicle (NEV) development in China has amounted to hundreds of billions of euros. An analysis of NEV by the European Commission estimates that between 2015 and 2020, China spent USD 60 billion (EUR 51 billion) on subsidies alone for the development of an EV industry and the uptake of NEV by the public (Pelkonen 2018). Research and development in the NEV landscape are estimated to have exceeded EUR 2 billion in the same period Chinese government and industry have also made substantial investment in the mining sector overseas. For example, in 2018, China controlled about 7% of the total value of African mine production, with much greater shares for some metals (cobalt more than 41%; copper about 28%) (Ericsson et al. 2020). China has also provided loans to African governments and state-owned enterprises totalling nearly USD 152 billion between 2000 and 2018 (Ericsson et al. 2020). These actions, together with vertical integration of the battery metals sector, have allowed China to gain control over raw material supplies and the battery market as whole, while adhering to their planned schedule for EV manufacture.

As highlighted in the American Mineral Security Act (Senate—Energy and Natural Resources 2019), there is an urgent need to develop people’s skills and knowledge across all stages of the supply chains of technology metals. This requires national programmes of education and training underpinned by government funding and delivered by the academic community and industry. The current dearth of such skills across the globe could lead to serious delays in procuring adequate and sustainable supplies of the metals needed for electrifying the automotive sector. In addition, the research community should work closely with industry to improve the knowledge base for technology metals and to develop innovative techniques to optimise their discovery, processing, use and recycling. The availability of researchers and industry personnel with relevant skills and knowledge will be key to the attainment of these objectives.

## Conclusions

Demand projections for battery raw materials suggest that supply will have to increase rapidly up to 2030. This raises the question of how that deadline can be met without compromising the EV production targets of governments and OEMs.

We have developed an indicative timeline for the entire EV supply chain from deposit identification to mining, refining and manufacturing. We have focussed on the raw material supply stages in tier 4 of the supply chain. Our analysis suggests an average of 8–11 years to identify a mineral reserve, a further 9–12 years for mine development and at least 10 years for tier 1–3 activities. Overall, therefore, the length of the timeline is likely to be in the range of 27 to 33 years. Consequently, projects in tier 3 and 4 should have been initiated around the start of the millennium in order to ensure their delivery by 2030. This clearly has not happened and we now urgently need to compress the timescale significantly to ensure that material supply does not become a serious constraint on EV production.

There are numerous challenges to be overcome if the timescale is to be shortened. These are of a diverse nature and relate to various technical, financial, environmental, social and governance issues. Analysis of these potential barriers allows various options for mitigating supply to be identified (Table 1). The quick wins to shortening the timescale of tier 4 of the EV supply chain include the following: the efficient governance of mineral resources through effective and holistic permitting and regulatory processes; the upscaling of existing or historic projects that already have some underpinning data and knowledge of the mineral resource; and the availability of high-quality data that can facilitate the identification of mineral targets. However, most of these measures are unlikely to have significant effects in the short term; rather, they should be viewed as contributing to an integrated strategic programme in which both governments and industry have essential roles to ensure adequate and sustainable raw material supply in the medium and long term.

Priority actions to mitigate raw material supply barriers include:

- supply chain convergence and increased ownership of the EV supply chain by OEMs investing in mining and mineral processing projects.
- government initiatives and alliances to diversify the supply base, improve supply chain resilience and ensure high standards of environmental performance.
- joint participation of the global community in the resolution of environmental, social and governance (ESG) challenges. Such initiatives should provide financial support, promote improved data collection, capacity building and knowledge transfer and undertake systemic research to address key ESG issues.
- development of the skilled workforce required throughout the supply chain so that raw materials can be sourced, processed, used and recycled in an efficient, safe and sustainable manner.
- conduct of applied research to provide new data on all aspects of raw material demand and supply and to develop holistic methods to provide sustainable solutions to technical and non-technical challenges.

## Data Availability

All datasets used in this manuscript are available from our publicly available repositories at the British Geological Survey that are accessible from the MineralsUK website.

## Footnotes

1 Class I (greater than 99.8% Ni) nickel is the principal source of battery grade nickel. However, class II nickel (less than 99.8% Ni) dominates global nickel production.
